# Galectin-3 causes enteric neuronal loss in mice after left sided permanent middle cerebral artery occlusion, a model of stroke

**DOI:** 10.1038/srep32893

**Published:** 2016-09-09

**Authors:** Xiaowen Cheng, Antonio Boza-Serrano, Michelle Foldschak Turesson, Tomas Deierborg, Eva Ekblad, Ulrikke Voss

**Affiliations:** 1 Neurogastroenterology unit, Department of Experimental Medical Science, Lund University, Lund, Sweden; 2 Neuroinflammation unit, Department of Experimental Medical Science, Lund University, Lund, Sweden.

## Abstract

In addition to brain injury stroke patients often suffer gastrointestinal complications. Neuroimmune interactions involving galectin-3, released from microglia in the brain, mediates the post-stroke pro-inflammatory response. We investigated possible consequences of stroke on the enteric nervous system and the involvement of galectin-3. We show that permanent middle cerebral artery occlusion (pMCAO) induces loss of enteric neurons in ileum and colon in galectin-3^+/+^, but not in galectin-3^−/−^, mice. *In vitro* we show that serum from galectin-3^+/+^, but not from galectin-3^−/−^, mice subjected to pMCAO, caused loss of C57BL/6J myenteric neurons, while myenteric neurons derived from TLR4^−/−^ mice were unaffected. Further purified galectin-3 (10^−6^ M) caused loss of cultured C57BL/6J myenteric neurons. Inhibitors of transforming growth factor β-activated kinase 1 (TAK1) or AMP activated kinase (AMPK) counteracted both the purified galectin-3 and the galectin-3^+/+^ pMCAO serum-induced loss *in vitro*. Combined we show that stroke (pMCAO) triggers central and peripheral galectin-3 release causing enteric neuronal loss through a TLR4 mediated mechanism involving TAK1 and AMPK. Galectin-3 is suggested a target for treatment of post-stroke complications.

Ischemic cerebral stroke is a major cause of death worldwide and is at present a leading cause of adult mortality and disability^1^. In addition to the brain injury, stroke patients are at risk of a number of comorbidities including cardiac, pulmonary and gastrointestinal (GI) complications as well as infections and sepsis, all affecting recovery^2^. GI complications promote bacterial translocation and include dysphagia, dysmotility and colorectal dysfunction^2^, which rely on enteric nervous system (ENS) control. Experimental stroke models have revealed that rodents subjected to unilateral permanent middle cerebral artery occlusion (pMCAO) exhibit decreased GI motility, intestinal mucosa damage, increased levels of circulating ghrelin^3^, reduced T- and B cell counts in Peyer’s patches^4^, and bacterial translocation^5^. Whether neurological manifestation in the ENS post stroke occurs in parallel to that in the central nervous system (CNS) is unknown. Post stroke neuroimmune interactions involving microglia and recruited peripheral macrophages determine neurological outcome in the CNS^6^. Galectin-3 (gal-3) is an evolutionarily preserved beta-galactoside-binding protein with both pro- and anti-inflammatory properties^7^. Recently it was shown that gal-3 secreted from IBA-1- (marker of microglia and recruited peripheral macrophages) positive cells is an endogenous toll-like receptor 4 (TLR4) agonist leading to a sustained pro-inflammatory response^8^. Gal-3 show ubiquitous subcellular distribution with multiple intra- and extracellular effects^9^. In the GI tract gal-3 stabilizes cell-cell junctions and enables polarization in intestinal epithelial cells^10^,^11^. Furthermore, it has been associated with GI cancers^12^, as well as with Crohn’s disease and ulcerative colitis^13^. Elevated serum levels of gal-3 correlates with poor outcome after acute heart failure and high gal-3 values are observed in heart failure patients with a history of stroke^14^.

This study aims to investigate possible consequences of pMCAO on the ENS and the possible role of gal-3 in enteric neuropathy.

## Results

### pMCAO causes loss of enteric neurons in colon and ileum from gal-3^+/+^, but not gal-3^−/−^ mice

**Figure 1:**
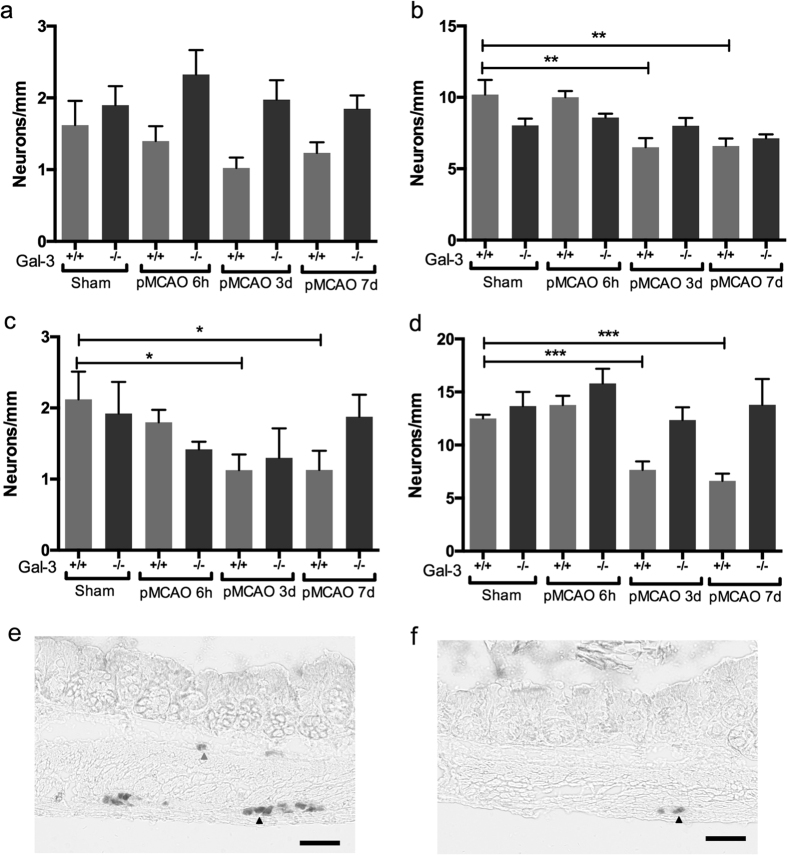
Gal-3^+/+^ (light grey bars), but not gal-3^−/−^ (dark grey bars), mice subjected to permanent middle cerebral artery occlusion (pMCAO) display significant losses of enteric neurons in ileum and colon. (**A**) In ileum no loss of submucous neurons is observed at any time point post pMCAO in either gal-3^+/+^ or gal-3^−/−^ mice. (**B**) Gal-3^+/+^, but not gal-3^−/−^, mice display significantly reduced numbers of myenteric neurons in ileum at 3 and 7 days, but not 6 hours, post pMCAO. (**C**) In colon of gal-3^+/+^ mice significant losses of submucous neurons are observed at 3 and 7 days, but not 6 hours, post stroke. pMCAO induces no neuronal loss in gal-3^−/−^ mice. (**D**) Gal-3^+/+^ mice display significant losses of myenteric neurons in colon 3 and 7 days, but not 6 hours, post stroke. No neuronal loss is noted in gal-3^−/−^ mice. (**E**,**F**) Representative micrographs of HuC/D immunostained colon sections from sham (**E**) and 3 days pMCAO (**F**) treated gal-3^+/+^ mice, illustrating loss of both submucous and myenteric neurons post pMCAO. Arrowheads indicate myenteric (black) and submucous (grey) neurons. Results are expressed as number of neurons per mm section, mean±SEM, (**A**,**B**) (ileum) sham gal-3^+/+^ n = 5, sham gal-3^−/−^ n = 3, 6 h gal-3^+/+^ n = 5, 6 h gal-3^−/−^ n = 4, 3d gal-3^+/+^ n = 4, 3d gal-3^−/−^ n = 4 and 7d gal-3^+/+^ n = 6, gal-3^−/−^ n = 5, (**C**,**D**) (colon) sham gal-3^+/+^ n = 10, sham gal-3^−/−^ n = 5, 6 h gal-3^+/+^ n = 5, 6 h gal-3^−/−^ n = 5, 3d gal-3^+/+^ n = 11, 3d gal-3^−/−^ n = 5, 7d gal-3^+/+^ n = 10 and 7d gal-3^−/−^ n = 4, * P < 0.05, **P < 0.01, ***P < 0.001, bar represents 50 μm.

The brain damage caused by pMCAO in gal-3^+/+^ and gal-3^−/−^ mice was assed in a separate study (Deierborg *et al*., to be published). A 15 mm^3^ barrel cortex lesion and a transient postsurgical weakness of both left and right front paws was found in both genotypes, in accord with previous reports^15^. At autopsy, the visceral organs were inspected and no lesions or abnormalities were identified. No differences between the sham groups in neither ileum nor colon were detected in the morphometric analyses or in the neuronal cell counting therefore sham mice were pooled into gal-3^+/+^ sham and gal-3^−/−^ sham groups. In ileum, no differences between the numbers of submucous neurons in pMCAO and sham operated mice were detected in either gal-3^+/+^ or gal-3^−/−^ groups (Fig. 1A). Gal-3^+/+^ mice displayed significantly reduced numbers of myenteric neurons at 3 (P < 0.01) and 7 (P < 0.01) days after pMCAO, compared to both sham and 6 h pMCAO (Fig. 1B). Gal-3^−/−^ mice displayed no loss of myenteric neurons at any time point after pMCAO compared to sham mice. In colon no significant differences in numbers of submucous neurons were detected in gal-3^−/−^ mice, regardless of treatment, but significant losses of submucous neurons were detected in gal-3^+/+^ mice 3 (P < 0.05) and 7 (P < 0.05) days, but not 6 hours, post pMCAO compared to the gal^+/+^ sham group (Fig. 1C). Also in colonic myenteric ganglia no significant losses were found in pMCAO gal-3^−/−^ mice (all time points), but significant losses of myenteric neurons were observed in gal-3^+/+^ mice 3 (P < 0.001) and 7 (P < 0.001) days, but not 6 hours, post pMCAO (Fig. 1D). Intestinal morphometry revealed transient thickening of ileal muscularis propria in gal-3^+/+^ mice 6 h post pMCAO in ileum and in colon mucosa 3 days post pMCAO. Results are summarized in Table 1.

### Sera from pMCAO treated gal-3^+/+^, but not from gal-3^−/−^, mice and purified gal-3 induce loss of myenteric neurons *in vitro*

**Figure 2:**
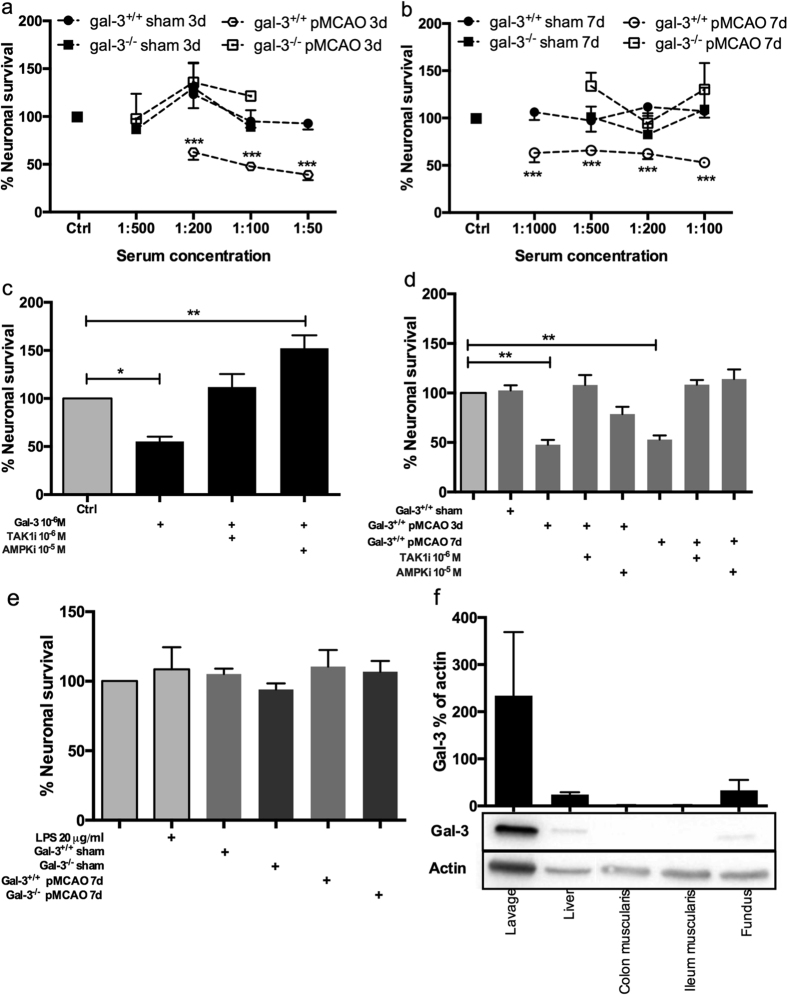
Sera from permanent middle cerebral artery occlusion (pMCAO) treated gal-3^+/+^ but not gal-3^−/−^, mice cause myenteric neuronal loss *in vitro*. (**A**,**B**) Gal-3^+/+^ (black circles) and Gal-3^−/−^ (black squares) sera from 3 (**A**) and 7 days (**B**) sham mice do not affect myenteric neuronal survival in C57BL/6 derived cultures. Neither do 3 (**A**) or 7 days (**B**) pMCAO sera from gal-3^−/−^ mice (open squares), while 3 (**A**) and 7 days (**B**) pMCAO sera from gal-3^+/+^ mice (open circles) significantly reduce myenteric neuronal survival in C57BL/6 derived cultures. (**C**) Purified gal-3 (10^−6^ M) induces myenteric neuronal loss in C57BL/6 derived cultures. This was prevented by the presence of inhibitors of TAK1 (TAK1i, 10^−6^ M) or AMPK (AMPKi, 10^−5^ M). (**D**) The gal-3^+/+^ pMCAO serum (1:100, 3 and 7 days post surgery) -induced neuronal loss could be prevented by presence of inhibitors of TAK1 (TAK1i, 10^−6^ M) or AMPK (AMPKi, 10^−5^ M). (**E**) Neither LPS nor sera (1:200) from sham or pMCAO 3 and 7 days treated gal-3^+/+^ and gal-3^−/−^ mice affect survival of myenteric neuronal in TLR4^−/−^ derived cultures. (**F**) Western blot analysis of gal-3 protein levels in immune cells collected by peritoneal lavage and digestive organs. Results are expressed as % neuronal survival compared to controls run in parallel (**A**–**E**) or as % gal-3 of actin (**F**), in mean ± SEM, n = 3–18, * P < 0.05, **P < 0.01, ***P < 0.001.

To test if the enteric neuronal losses noted *in vivo* post pMCAO were mediated by way of circulating factors, sera from sham and pMCAO treated animals were added to primary cultures of myenteric neurons, derived from C57BL/6J mice. Control wells displayed 2.5 ± 0.2 neurons/mm^2^ (n = 78) of evenly distributed neurons, with a varicose fibre network. No significant changes in neuronal survival were observed between controls and cultures treated with 3 (Fig. 2A) or 7 (Fig. 2B) days sham sera at any concentration tested and results were pooled into ctrl, gal-3^+/+^ sham and gal-3^−/−^ sham. Exposure with pMCAO serum from gal-3^−/−^ mice did not change neuronal survival in any of the concentrations tested. Exposure of serum from pMCAO treated gal-3^+/+^ mice 3 (P < 0.001) and 7 (P < 0.001) days post pMCAO, caused a significant neuronal loss. Results are summarized in Fig. 2A,B. Since gal-3 in the CNS is able to mediate both pro- and anti-inflammatory responses in response to injury^16^,^17^,^18^, we investigated the effect of purified gal-3 (10^−6^ M) on cultured enteric neurons, and found a significant loss (P < 0.05) (Fig. 2C).

### Inhibition of TAK1 or AMPK prevent purified gal-3- and gal-3^+/+^ pMCAO serum-induced myenteric neuronal loss *in vitro*, through mechanisms involving TLR4 but not LPS

Gal-3 has recently been put forward as a TLR4 ligand, potentiating lipopolysaccharide (LPS) mediated TLR4 activation^8^. The intestinal barrier is likely to be compromised post stroke^3^,^4^,^5^, enabling bacterial translocation and concomitant LPS efflux. LPS exposure to enteric neurons causes neuronal loss through mechanisms involving transforming growth factor &beta - activated kinase 1 (TAK1) and AMP activated kinase (AMPK)^19^. Both purified gal-3 (10^−6^ M)- and pMCAO gal-3^+/+^ (1:100) serum-induced neuronal losses were counteracted by the simultaneous addition of TAK1 inhibitor (10^−6^ M) or AMPK inhibitor (10^−5^ M) (Fig. 2C,D). Notable is that serum analysis for endotoxin/LPS levels using the LAL assay showed low levels (0.03–0.05 EU/ml) and no pMCAO-induced increase compared to sham operated animals, regardless of genotype. To investigate possible activation of TLR4 in the pMCAO serum-induced loss of myenteric neurons TLR4^−/−^ derived cultures were exposed to sham (1:200) or pMCAO serum (1:200). Control wells from cultures of TLR4^−/−^ mice displayed 1.9±0.3 neurons/mm^2^ (n = 16) of evenly distributed neurons, with varicose fibre networks. In contrast to C57BL/6J derived neuronal cultures^19^, LPS (20 μg/ml) did not lead to neuronal loss confirming genotype. Further exposure to 7 days pMCAO serum from gal^+/+^ mice did not induce neuronal loss in TLR4^−/−^ cultures; this is in contrast to C57BL/6J derived cultures (Fig. 2E).

### Gal-3 is highly expressed in peripheral immune cells

Western blot analysis of tissues from digestive organs and immune cells from peritoneal lavage were conducted to determine the possible source of gal-3. Western blot revealed high levels of gal-3 in peripheral immune cells and low levels in digestive organs (Fig. 2F).

## Discussion

Stroke triggers an inflammatory cascade caused by *e.g.* reactive oxygen or nitrogen species and damage-associated molecular pattern (DAMP) signals released from neurons, glia, microglia and vascular cells in the affected area. DAMP signals mediate inflammatory responses utilizing the same pattern recognition receptor (PRR) system as pathogens^20^. This concept of *sterile inflammation* highlights the ability of the immune system to sense and react to internal as well as external clues^20^,^21^. In the hours, days and weeks after cerebral ischemia a peripheral immune response involving both innate and adaptive responses are present in blood with elevated levels of monocytes, activated- and regulatory T cells, and cytokines such as TNF, IL1β and IL6^20^,^22^,^23^,^24^. Possible enteric comorbidity or neuropathy in response to cerebral ischemia or the accompanying post stroke inflammation have not previously been investigated. In the current study we also investigated if gal-3 in the periphery, like in the central nervous system, is a mediator of the pro-inflammatory response post stroke.

In both gal-3^+/+^ and gal-3^−/−^ mice pMCAO, a model of focal ischemic stroke, induces brain injury equal in volume (Deierborg, unpublished). Notable is that pMCAO in gal-3^+/+^ mice induces loss of enteric neurons in both ileum and colon. This was evident after 3 days and was not exaggerated further at 7 days. In colon both submucous and myenteric ganglia were affected after the pMCAO insult, while only myenteric neurons were lost in ileum. The myenteric neuronal loss in colon reached 60% compared to 40% in ileum suggesting a proximo-distal gradient in neuronal susceptibility to pMCAO. No pMCAO-induced neuronal loss was however, noted in intestines of mice lacking the gal-3 gene. Loss of neurons may reflect intestinal atrophy^25^, however morphometric measures showed only minor transient thickenings of mucosa and muscularis propria suggesting the neuronal loss is not a consequence of intestinal inactivity but a primary event due to pMCAO. We further showed, *in vitro*, that serum from gal-3^+/+^, but not from gal-3^−/−^, mice subjected to pMCAO, induced loss of cultured myenteric neurons. Also exposure to purified gal-3 induced loss of myenteric neurons *in vitro*. Both gal-3 *per se* and gal-3^+/+^ pMCAO serum-induced neuronal losses could be prevented by the presence of either the TAK1 inhibitor or the AMPK inhibitor. We have previously shown that LPS-induced and TLR4 activated loss of myenteric neurons *in vitro* depend on this pathway^19^. Moreover, in contrast to C57BL/6J derived cultures, serum from gal-3^+/+^ pMCAO mice did not affect neuronal survival in myenteric cultures from TLR4^−/−^ mice. These findings suggest that the mechanism behind pMCAO-induced enteric neuronal loss is by way of gal-3 triggered activation of TLR4. Together our data strongly suggest that focal ischemic stroke causes enteric neuronal loss, in parallel with central manifestations, and that the neuroimmune mechanism behind involves gal-3 and TLR4 receptor activation. This may explain the graded and extended (involving both submucous and myenteric neurons) neuronal susceptibility to pMCAO observed *in vivo*, as immunocytochemical analyses show that TLR4 is more abundantly expressed in the distal, compared to the proximal, bowel in both neurons and glia cells^26^,^27^. Astrocytes mediate a neurotoxic inflammatory response in the central nervous system through TLR4 activation causing oxidative stress^28^. As TLR4 is expressed in both neurons and glia in the ENS a toxic contribution through reactive oxygen species can’t be entirely excluded. Regardless, gal-3 is revealed a key mediator of pMCAO-induced enteric neuropathy. This suggestion is further strengthened by our finding that the neuronal loss reached a plateau at 3 days, coinciding with the peak of gal-3 expression in microglia and recruited macrophages in the brain^29^. In addition analysis of gal-3 showed high levels of gal-3 in peritoneal immune cells.

Gal-3 is able to form stable complexes with LPS, potentiating LPS ability to activate immune cells such as neutrophils^9^. A similar mode of action could be at play in neurons, however analysis of sera from gal-3^+/+^ and gal-3^−/−^ sham as well as pMCAO mice showed very low LPS levels across all groups and genotypes. Although unable to entirely dismiss LPS as a possible player in pMCAO-induced enteric neuronal loss *in vivo*, our data strongly suggests gal-3 to be the main mediator behind the enteric neuronal loss. Gal-3 acts as an endogenous TLR4 agonist able to, independent of LPS, mediate a cellular response on microglia^8^, and neutrophils^9^. Inhibition or genetic removal of TLR4 is neuroprotective in models of cerebral ischemia, highlighting a central role of TLR4 in neuroinflammation^30^,^31^,^32^. As shown in present study this extends also to enteric neurons, as myenteric cultures from TLR4^−/−^ mice were resistant to gal-3^+/+^ pMCAO serum exposure. Interestingly both TAK1 and AMPK inhibition protect central^33^,^34^, and enteric neurons after cerebral ischemic damage suggesting common pathways activated.

In conclusion a cerebral ischemic event like stroke, modelled by pMCAO, causes neuroinflammation leading to a significant loss of enteric neurons. The enteric neuronal loss is targeted through a gal-3 mediated TLR4 activation independent of LPS. This finding highlights the presence of parallel manifestations in central and enteric nervous systems post stroke, involving neuroimmune interactions, gal-3 induced TLR4 activation and the TAK1/AMPK pathway. This is the first report showing the existence of a neuroinflammatory response emanating in the central and transmitted to the peripheral i.e. enteric nervous system.

## Methods and Material

### Ethical statement

Procedures were approved by the regional Malmö/Lund committee for experimental animal ethics, Swedish board of Agriculture (M301-09 and M95-15). Animals were used in accordance with the European Community Council Directive (2010/63/EU) and the Swedish Animal Welfare Act (SFS 1988:534).

### Animals

Gal-3^+/−^ mice (35; C57BL/6 background), were obtained from Dr. K. Sävman, Gothenburg University and housed and bred at the centre of Production and Animal Experimentation Lund University. Genotyping was as described in Doverhag *et al*.^36^. Gal-3^−/−^ mice exhibit normal behaviour and normal reproductive capacities compared with gal-3^+/+^ mice^37^. Male C57BL/6J (n = 26, 25–30 g, Janvier labs, FR) and TLR4^−/−^ (n = 4, 20–28 g^38^, kind gift from Professor C. Svanborg, Lund University) were used for *in vitro* experimentation. Male C57BL/6J (n = 6, 23–30 g, Janvier labs, FR) were used for collecting tissues for western blot analyses of gal-3.

### *In vivo* experiments

Gal-3^+/+^ and gal-3^−/−^ littermates were subjected to either permanent middle cerebral artery occlusion (pMCAO, gal-3^+/+^ n = 29, gal-3^−/−^ n = 19) by permanently occluding the distal part of the left middle cerebral artery as previously described or sham (gal-3^+/+^ n = 11, gal-3^−/−^ n = 6) surgery^15^. At selected time points post surgery mice were anesthetised (Ketalar, Rompun) and either killed by cervical dislocation or guillotine. pMCAO animals were sacrificed 6 hours (gal-3^+/+^ n = 6, gal-3^−/−^ n = 6), 3 days (gal-3^+/+^ n = 12, gal-3^−/−^ n = 11) and 7 days (gal-3^+/+^ n = 10, gal-3^−/−^ n = 5) after surgery, sham animals were scarified 3 days (gal-3^+/+^ n = 7, gal-3^−/−^ n = 3) and 7 days (gal-3^+/+^ n = 4, gal-3^−/−^ n = 3) after surgery. Serum for *in vitro* testing was sampled from guillotined mice 3 and 7 days after pMCAO or sham operation (n = 3 in each group). Collected blood was centrifuged 5 min at 1000 rcf, serum collected and pooled in respective treatment groups. Aliquots (20 μL) were stored at −80 °C until use. At autopsy the abdomen was opened and internal organs inspected. The intestines were removed and fixed in 4% paraformaldehyde in 0.1 M phosphate buffer overnight at 4 °C and rinsed in Tyrode’s solution containing 10% sucrose three times before segments of ileum and colon were orientated and mounted for longitudinal and cross sectioning in FSC 22^®^ Clear (Leica Biosystems, SE), frozen on dry ice and sectioned (10 μm). The sections were processed for immunocytochemistry and histochemistry.

In separate experiments C57BL/6J mice were anesthetised (Ketalar, Rompun) and liver, fundus, ileum and colon were collected. Animals were killed by heart puncture. Muscularis propria was further separated from ileum and colon and samples were snap frozen in liquid nitrogen. Intestinal lavage was performed on C57BL/6J mice killed by cervical dislocation, 5 ml saline containing 5U heparin per ml were injected into the peritoneal cavity, followed by 5 min abdominal massage. The immune cell rich fluid (2.5 ml) was removed and centrifuged at 700 rcf for 3 min at room temperature, prior to protein extraction.

### *In vitro* experiments

#### Primary myenteric neuronal cultures

Myenteric neurons were dissociated from mouse small intestine, using a previously described method^19^,^39^. Cell cultures were prepared by adding 50 μL of a constantly mixed cell suspension into 8-well chambers (30108, SPL, Saveen Werner, SE) prefilled with 450 μL of Neurobasal A (NBA) cell culture medium (NBA, Thermo Fisher Scientific, SE with 10%v/v fetal bovine serum FBS, BioWest, FR, 0.5 mM L-glutamine K0282 and 50 U/mL penicillin, 50 μg/mL streptomycin A2213, BioChrom AG). From each animal two 8-well chambers (69 mm^2^/well) were prepared. Fresh medium containing applicable experimental test agents was added to cultures after a 4 day equilibration culture period. Control wells were cultured in parallel. Cells were fixed in Stefanini’s fixative, rinsed in Tyrode solution, frozen, thawed and subjected to immunocytochemistry.

#### Material and experimental treatments

Stock solutions of 6-[4-(2-Piperidin-1-ylethoxy)phenyl]-3-pyridin-4-ylpyrazolo[1,5-a]pyrimidine (compound C, AMPKi, Merck, SE) an inhibitor of AMP-activated kinase (AMPK), (5Z)-7-Oxozeaenol (TAK1i, Tocris Bioscience, UK) an inhibitor of transforming growth factor β-activated kinase 1 (TAK1) and *Escherichia coli* serotype O111:B4 derived LPS (Merck, SE) were prepared according to manufacturer’s recommendations, aliquoted and stored at −20 °C. Human purified gal-3 (kind gift Professor H. Leffler, Lund University) was aliqouted in PBS and stored at −20 °C. Pooled serum samples containing serum from 1. gal-3^+/+^ sham 3 days (n = 3), 2. gal-3^+/+^ sham 7 days (n = 3), 3. gal-3^+/+^ pMCAO 3 days (n = 3), 4. gal-3^+/+^ pMCAO 7 days (n = 3), 5. gal-3^−/−^ sham 3 days (n = 3), 6. gal-3^−/−^ sham 7 days (n = 3), 7. gal-3^−/−^ pMCAO 3 days (n = 3), 8. gal-3^−/−^ pMCAO 7 days (n = 3) were stored at −80 °C.

Various sets of experiments were performed. Incubations were for 4 days, with untreated controls always run in parallel. C57BL/6J derived cultures were exposed to 1. Serum from gal-3^+/+^ animals (1:1000-1:50, samples 1-4), 2. Serum from gal-3^−/−^ animals (1:500-1:100, samples 5-8), 3. Human gal-3 (10^−6^ M) with or without TAK1i (10^−6^ M) or AMPKi (10^−5^ M)^19^, or 4. Serum from gal-3^+/+^ animals (1:100, sample 1-4) with or without TAK1i (10^−6^ M) or AMPKi (10^−5^ M). TLR4^−/−^ derived cultures were exposed to 1. Serum from gal-3^+/+^ animals (1:200, sample 1,2 and 4), 2. Serum from gal-3^−/−^ animals (1:200, sample 5, 6 and 8) or 3. LPS (20 μg/ml)^19^.

### Immunocytochemistry and histochemistry

For analysis on neuronal survival, cryosections were subjected to antigen retrieval by microwaving 2 × 8 min at 650 W in citrate acid buffer (0.01 M, pH 6). After cooling they were washed 20 min in running tap water, 10 min in 3% hydrogen peroxide (H_2_O_2_) and 10 min in PBS with 0.25% Triton X-100 (PBS-T). Sections were exposed to primary antibodies against human neuronal protein HuC/D (A-21271, Molecular Probes, Thermo Fisher Scientific, SE; dilution 1:400^40^) at 4 °C overnight, followed by anti-mouse IgG SignalStain® Boost IHC detection reagent (#8125, Cell signaling), and 3,3′-diaminobenzidine (DAB) peroxidase (HRP) staining (#SK4100, Vector laboratories). Morphometric analyses were on toluidine blue (0.01% in 60% ethanol) stained cryosections.

Cultures were immunolabeled with primary antibodies against human protein gene product 9.5 (PGP 9.5, Ultraclone, UK; dilution 1:1200^41^), incubated overnight in moist chambers at 4 °C followed by incubation 1 h at RT with secondary antibodies (Alexa Fluor 594 donkey anti rabbit, 711-585-152, Jackson Lab Inc, USA). Hoechst (Thermo Fisher Scientific, SE) cell nuclei counter staining was performed according to manufacturer’s protocol. Mounting was in PBS:glycerol 1:1.

### Endotoxin assay

Pooled serum samples 1-8 were analysed by Limulus amoebocyte lysate (LAL) assay for endotoxin levels with a commercially available kit (Pierce LAL chromogenic Endotoxin Quantification Kit, Thermo Fisher Scientific, SE) used according to the manufacturer’s protocol.

### Western blot

Total protein was extracted in RIPA buffer (Merck, SE) containing a soluble protease inhibitor cocktail (1 tablet per 50 mL RIPA buffer according to manufacturer’s protocol, Thermo Fisher Scientific, SE). Protein concentrations were estimated using the Bradford Protein Quantification method (BCA Protein Assay KIT, Thermo Fisher Scientific, SE). Western blot analysis for gal-3 (M3-38) was performed using 10 μg protein extract separated on pre-cast 4–20% SDS-PAGE gels (Stain-Free Gels, BioRad, SE). Precision Plus Protein Western Blot pre-stained standard (BioRad, SE) was used as molecular weight marker. Densitometry was by ChemiDoc XRS+ and Image Lab, both from BioRad analysis software.

### Analysis

Submucous and myenteric neurons were counted in longitudinally cut sections from ileum and colon, using a computerized image analysing system (NDP view 2, Hamamatsu, JP). Sections comprised at least a total length of 15 mm cut at 3-5 different depths, per region and mouse. Results are expressed as numbers of HuC/D-immunoreactive (IR) submucous or myenteric neurons per mm section. Heights of mucosa and muscularis propria were measured on toluidine blue stained sections using mean values of 8-10 representative measurements from each region of each mouse. Survival of cultured neurons was calculated by counting the total number of PGP9.5-IR neurons within the wells using fluorescence microscopy (Olympus BX43, LRI, SE) with appropriate filter setting and expressed as percentage of the control well run in parallel.

LAL contents in serum samples 1-8 were plotted against a LPS standard curve using non-linearly fit.

### Statistical analysis

Data are presented as means ± SEM and analysed by GraphPad Prism (GraphPad Software Inc, USA). In the experimental groups *in vivo* n = 3–11 animals and in the groups *in vitro* n = 3–18 repeats from a minimum of 3 different animals. Statistical significance was for *in vivo* data determined using two-way analysis of variance (ANOVA) and for *in vitro* data one-way ANOVA. These were followed by Dunnet’s post hoc test towards the sham group (*in vivo* data) or untreated controls (*in vitro* data). T-test was used to determine differences between sham groups. A confidence level of 95% was considered significant.

## Additional Information

**How to cite this article**: Cheng, X. *et al*. Galectin-3 causes enteric neuronal loss in mice after left sided permanent middle cerebral artery occlusion, a model of stroke. *Sci. Rep.* **6**, 32893; doi: 10.1038/srep32893 (2016).

## Figures and Tables

**Table 1:** Morphometric analyses of ileum and colon, results presented as mean ± SEM, *p < 0.05 compared to sham.

Genotype	Treatment	IIeum mucosa, μm	Ileum muscularis propria, μm	Colon mucosa, μm	Colon muscularis propria, μm
Gal-3^+/+^	Sham	309 ± 6	43 ± 6	119 ± 14	63 ± 7
	6 h pMCAO	350 ± 11	64 ± 3*	135 ± 12	77 ± 4
	3d pMCAO	299 ± 17	45 ± 1	198 ± 24*	80 ± 5
	7d pMCAO	370 ± 29	54 ± 8	132 ± 13	85 ± 11
Gal-3^−/−^	Sham	273 ± 19	49 ± 7	111 ± 8	69 ± 3
	6 h pMCAO	248 ± 32	55 ± 2	176 ± 42	103 ± 11
	3d pMCAO	269 ± 28	45 ± 6	178 ± 40	92 ± 13
	7d pMCAO	279 ± 26	55 ± 10	161 ± 31	85 ± 8
